# Supplementary figures and images for: A Pathogenic Variant Reclassified to the Pseudogene PMS2P1 in a Patient with Suspected Hereditary Cancer
Source: Int J Mol Sci. 2023 Jan 11;24(2):1398. doi: 10.3390/ijms24021398 (PMC9864156; doi:10.3390/ijms24021398)

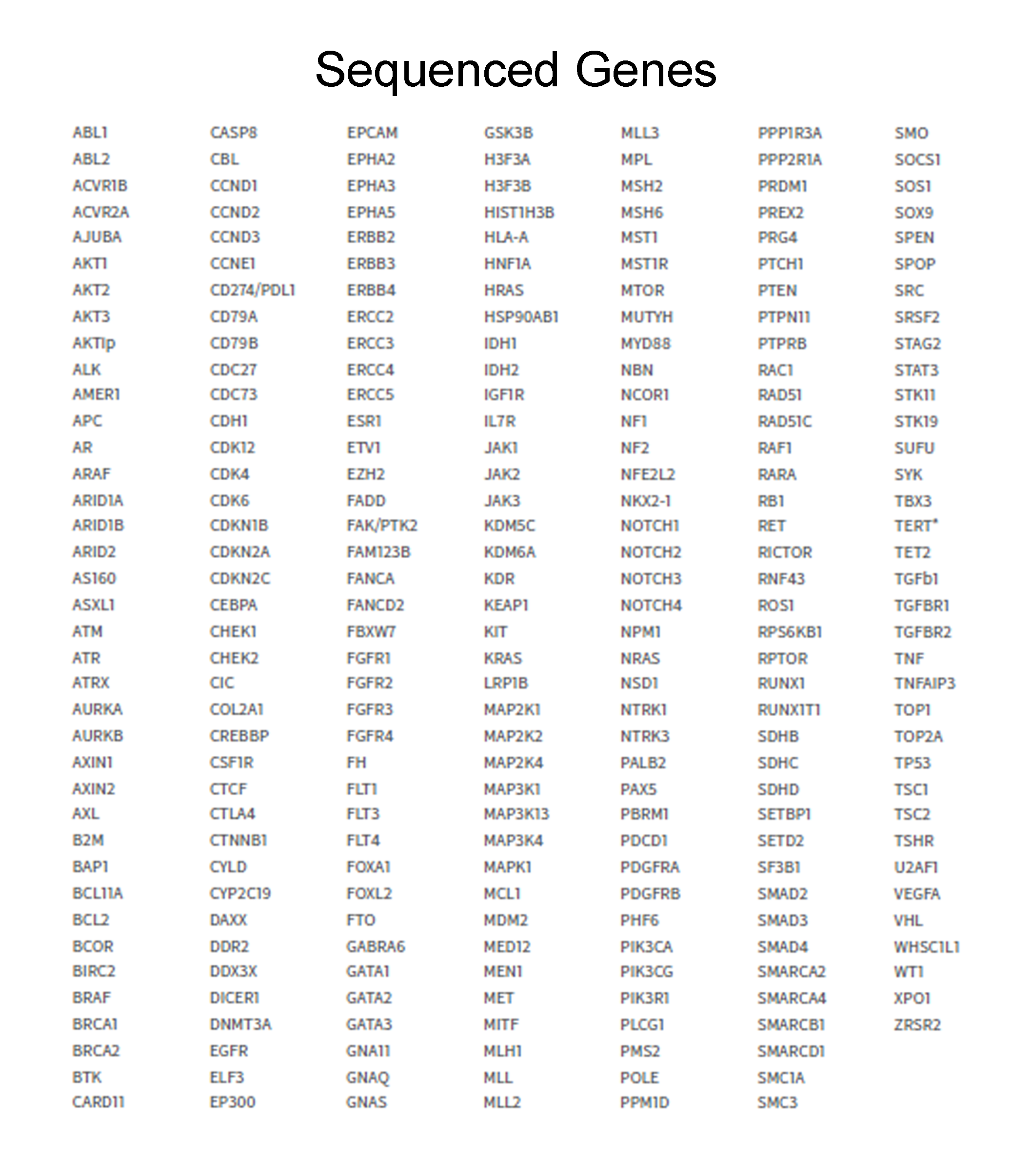

Supplement: Supplementary file 1 [file ijms-24-01398-s001.zip › Supplementary_S1 response.tif]

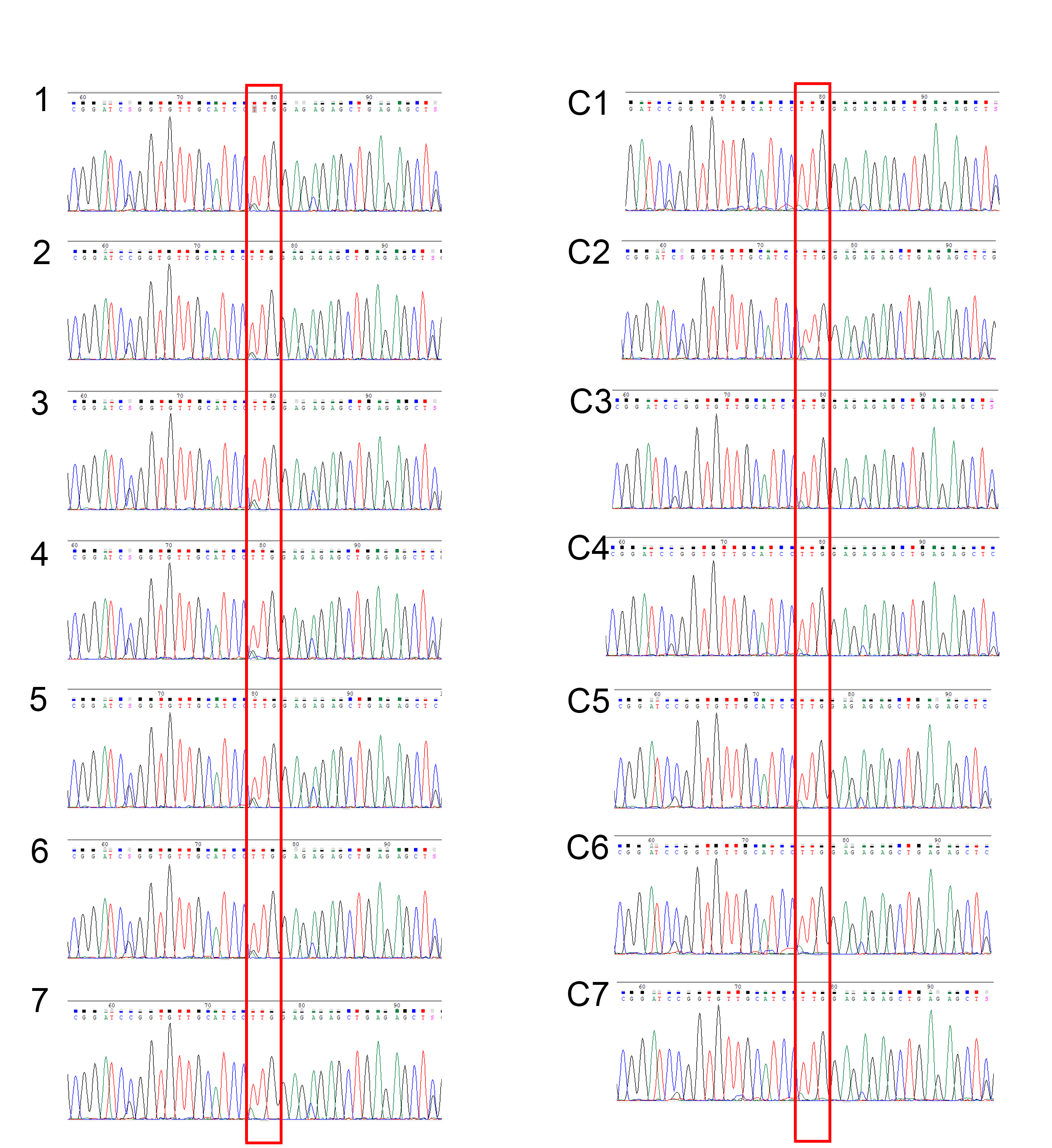

Supplement: Supplementary file 1 [file ijms-24-01398-s001.zip › Supplementary_S2.tif]

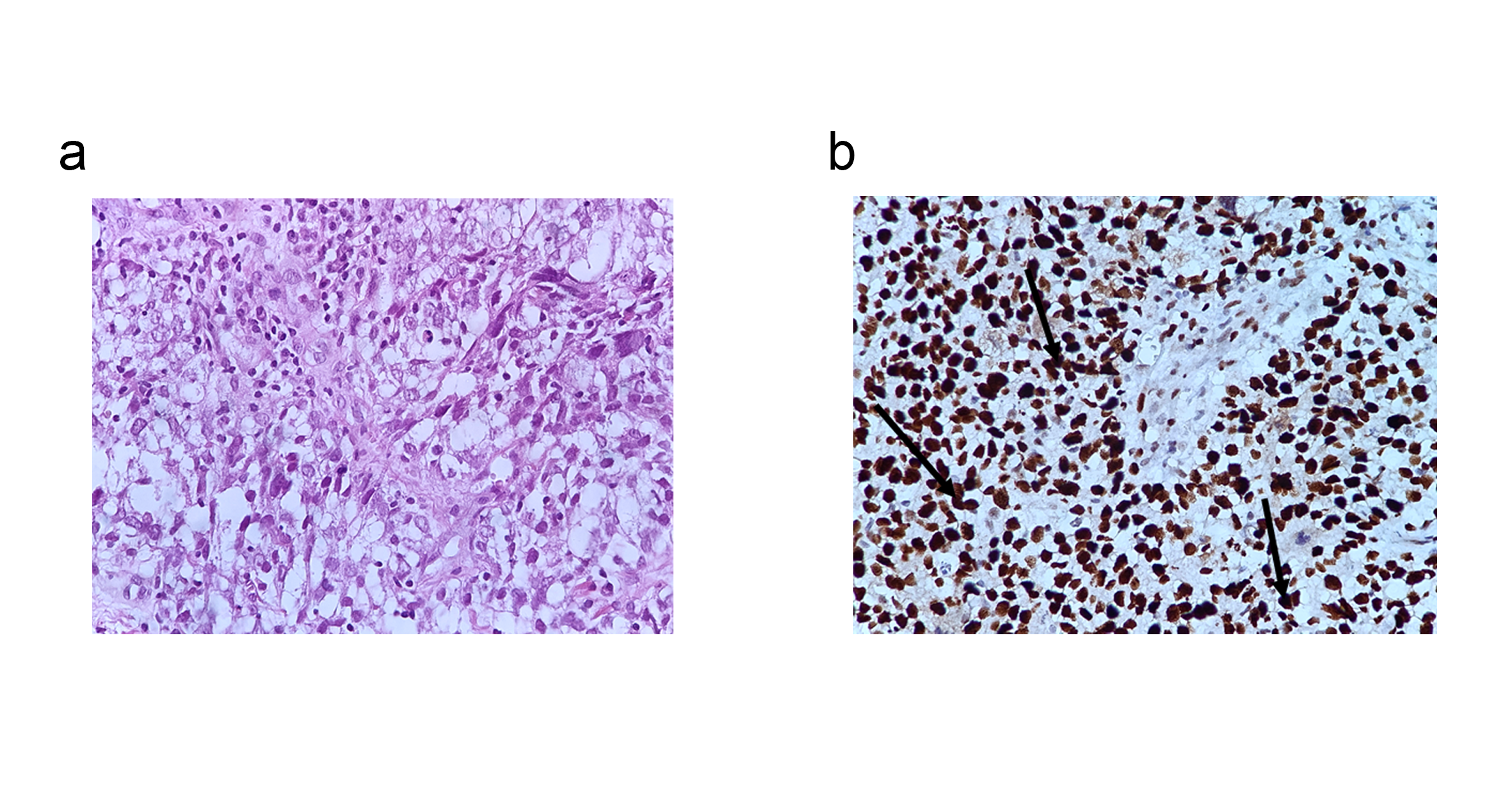

Supplement: Supplementary file 1 [file ijms-24-01398-s001.zip › Supplementary_S3.tif]
